# A massively asynchronous, parallel brain

**DOI:** 10.1098/rstb.2014.0174

**Published:** 2015-05-19

**Authors:** Semir Zeki

**Affiliations:** Laboratory of Neurobiology, University College London, London WC1E 6BT, UK

**Keywords:** parallel processing, hierarchical processing, asynchronous brain operations, perceptual asynchrony, Riddoch Syndrome

## Abstract

Whether the visual brain uses a parallel or a serial, hierarchical, strategy to process visual signals, the end result appears to be that different attributes of the visual scene are perceived asynchronously—with colour leading form (orientation) by 40 ms and direction of motion by about 80 ms. Whatever the neural root of this asynchrony, it creates a problem that has not been properly addressed, namely how visual attributes that are perceived asynchronously over brief time windows after stimulus onset are bound together in the longer term to give us a unified experience of the visual world, in which all attributes are apparently seen in perfect registration. In this review, I suggest that there is no central neural clock in the (visual) brain that synchronizes the activity of different processing systems. More likely, activity in each of the parallel processing-perceptual systems of the visual brain is reset independently, making of the brain a massively asynchronous organ, just like the new generation of more efficient computers promise to be. Given the asynchronous operations of the brain, it is likely that the results of activities in the different processing-perceptual systems are not bound by physiological interactions between cells in the specialized visual areas, but post-perceptually, outside the visual brain.

## 1. Introduction

In a synchronous chip, the clock's rhythm must be slow enough to accommodate the slowest action in the chip's circuits … Even though many other circuits on that chip may be able to complete their operations in less time, these circuits must wait until the clock ticks again before proceeding to the next logical step. In contrast, each part of an asynchronous system takes as much or as little time for each action as it needs. Complex operations can take more time than average, and simple ones can take less. Actions can start as soon as the prerequisite actions are done, without waiting for the next tick of the clock. Thus, the system's speed depends on the average action time rather than the slowest action time. Sutherland & Ebergen [1, p. 64]

Many would today subscribe to the view that the visual brain is organized to process visual signals in parallel, by which I mean that different attributes such as colour, motion and form are processed by separate systems [2]. This is reflected anatomically in the parallel outputs from the primary visual cortex, area V1, to different, specialized, visual areas of the prestriate cortex [3,4]. As I discuss below, parallelism may be an even more pervasive strategy in the visual brain than is commonly assumed.

There are probably sound computational reasons for this neural separation of functions as well as more mundane perceptual ones [5]. The latter can be summarized as follows: that different attributes do not necessarily co-occur. A given colour can invest any form, and a form can be in any colour. If the specific form of a stimulus (or its direction of motion) always co-occurred with a specific colour, then the stimulus can be specified by any of these attributes. But, this is not the case. Moreover, there are different computational demands for processing different attributes, for example colour and motion. The former requires the brain to register the wavelength–energy composition of light coming from one surface and its surrounds simultaneously, whereas the latter requires it to register activity at least at two successive points in time.

Many would today also subscribe to the view that each one of these parallel systems consists of several hierarchical, apparently sequential, stages [3,4]. Some would go beyond and subscribe to a more extreme form of hierarchies, or rather the lack of it and the lack of a necessity for it as well. They would claim that cells along the visual pathways that process signals related to colour also process signals related to form as well as motion, in other words that they are ‘multiplex’ cells [6–8]. I do not agree with the latter view nor do I think that its proponents have made a near enough compelling case for it. But, this is not the place to go into a discussion of the relative merits of the different processing strategies proposed, because, whatever strategy one is inclined to prefer, each comes with a major problem, which is the same for all. The problem is that of perceptual asynchrony, namely that different attributes of the visual scene such as form, colour and motion are not perceived simultaneously. Instead, some attributes (such as colour) are perceived before others (such as motion), in the millisecond scale (see below).

Although largely restricted to a discussion of the visual brain, I believe that both the problem of perceptual asynchrony and what it reflects, namely that the operations of the brain are massively asynchronous with respect to each other, may be relevant to other sensory systems as well, and indeed, to higher cognitive processing systems in the brain. I suspect that there are lessons to be learned from this for developing future brain research strategies and possibly for computational neuroscience as well, although I am no expert in the latter field. I have not been exhaustive in citing the relevant literature, which is altogether enormous, but have selected instead papers with which I am familiar, many from our own work, and which not only provide good evidence in favour of both hierarchical and parallel processing systems, but also point to the problem associated with both. I also concentrate on the three visual attributes that I am most familiar with, namely colour, form and motion, but believe that these are sufficient to illustrate the general point that I am making, because they all point, ineluctably, to the brain as a massively asynchronous, parallel, organ. Hence, my emphasis in this review is more on the brain as an asynchronous organ, and the imperative to investigate it as such in future research.

## 2. The problem: asynchronous perception

**Figure 1.**
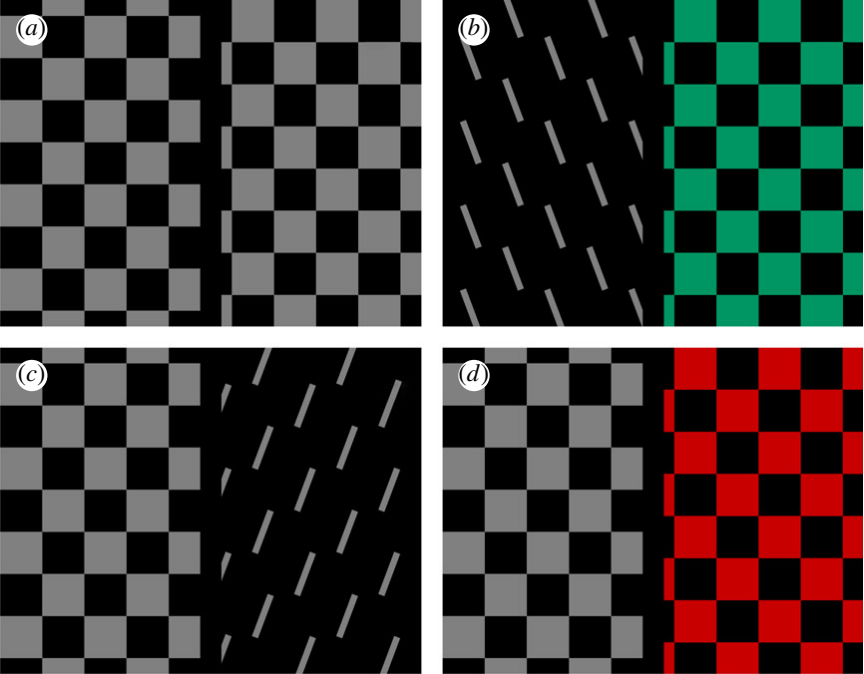
The perceptual pairing experiment, in which subjects view stimuli, each of which can be in one of two conditions, in two halves of the screen. Their task is to pair the condition in one half with that in the other. In (*a*), a checkerboard pattern moves up and down on the left hand, whereas the identical stimulus moves left–right on the right. The task for subjects here is to indicate what direction the motion of the pattern was on the left (left or right) when the one on the right was moving upwards. In (*b*), one of two orientations is presented on the left and one of two colours (green or red) is presented on the right. In (*c*), the pairing is between movement of the checkerbard (up–down or left–right) with one of two orientations, whereas in (*d*), the pairing is between colour and motion. (Adapted from Moutoussis & Zeki [10].)

**Figure 2.**
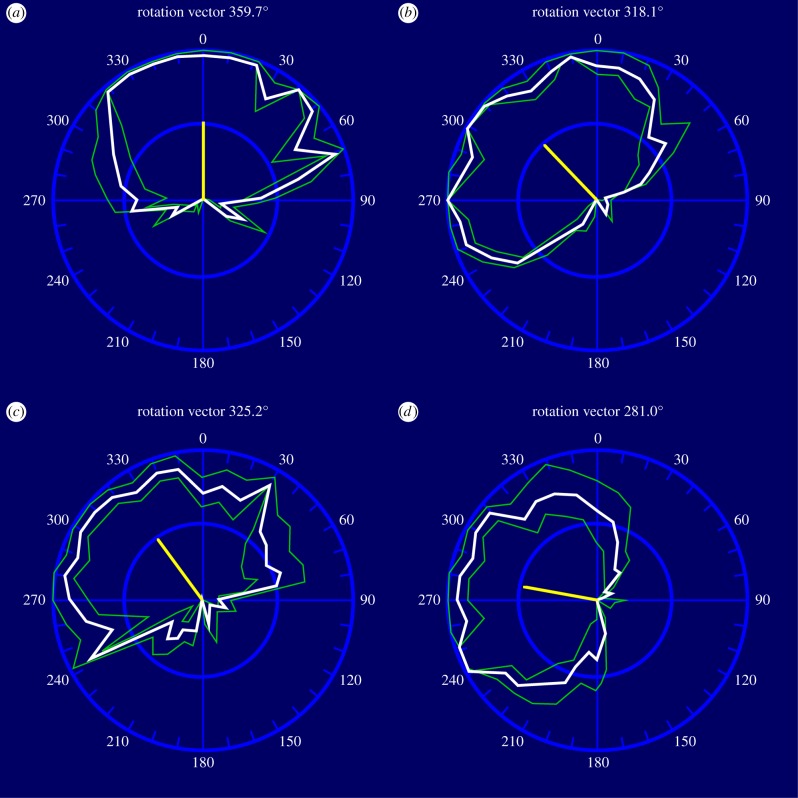
The averaged responses (in white), the standard deviations (in green) and the response vectors of four subjects to (*a*) up–down and left–right motion, (*b*) colour and orientation, (*c*) orientation and motion and (*d*) colour and motion pairings. (Adapted from Moutoussis & Zeki [10].)

A central problem in visual perception is that of perceptual asynchrony, demonstrated in psychophysical pairing experiments [9,10], in which subjects are asked to pair two states of two attributes (for a review, see Moutoussis [11]). The experiment has variants, but essentially is as follows: subjects have to pair one of two rapidly alternating colours (say red and green) which appear in one half of a monitor screen with one of two rapidly changing directions of motion (say up and down) of a checkerboard pattern, or one of two rapidly changing orientations, which appear in the other half of the screen (figure 1*d*). The stimuli, one on each half of the screen, oscillate between their two states, the oscillations on either side having the same period *T* but being presented at various phase differences with respect to each other. The experiment can equally well be done with a single stimulus, say a checkerboard pattern that moves up and down and changes in colour, the changes in direction of motion and colour being at different phases. The results are illustrated as polar (response) curves (figure 2). In these, the percentage of times that the answer was X for the property of the right half of the screen when Y is the property of the left half is plotted for each phase difference. If there is no difference in the time taken to perceive colour and motion, the response curves should be broadly similar and the vector of the curves vertical (figure 2*a*). On the other hand, if there is a difference in perception times for the two attributes, the response curve will deviate from the veridical one, in one direction or the other. In the colour–motion pairing experiment, for example, a clockwise deviation of the response curve indicates that motion is perceived first, and an anticlockwise deviation that colour is perceived first.

What these pairing experiments show is that these different attributes of a visual scene are not perceived simultaneously. Instead, colour is perceived before orientation (form) and orientation before motion [9,12–16], the perceptual asynchrony between colour and motion being as large—in neural terms—as 80 ms. The functional specialization of the visual brain thus appears to be projected in time. Moreover, because of this asynchrony, we misbind different attributes over very brief time windows, even though we bind them correctly over longer periods, something that has to be accounted for. In colour–motion pairing experiments, for example, subjects pair the direction of motion that was present on a screen at time *t*, with the colour that was present 80 ms earlier [9]. In other words, they misbind visual attributes that occur simultaneously in time (on a screen), which suggests that there is no system in the brain that ‘waits' for all the processing systems to terminate their tasks before binding them. Hence, to perceive colour and motion at the same identical moment, one would have to present the colour some 80 ms before the direction of motion, in objective time.

Perceptual asynchrony may not be evident in every psychophysical task employing colour and motion. There is some disagreement as to whether there is any temporal asynchrony in temporal order judgements (TOJ) in which subjects have to decide, after the presentation, the order of appearance of perceptual events [11], with some finding no asynchrony [17], whereas others maintain that colour changes precede, perceptually, motion changes occurring at the same time [13]. Even assuming that there is no perceptual asynchrony in TOJ experiments, one is inevitably led to the conclusion that, if the task is so critical, then the neural mechanisms underlying them are themselves asynchronous, because it implies that different tasks involving these two attributes take different times to completion.

Although perceptual asynchrony can only be demonstrated over very brief time windows lasting about 100 ms, it, nevertheless, offers us a window into the strategies that the brain uses to construct the visual image.

## 3. Asynchrony as a consequence of different times taken to process different attributes

One explanation given for asynchronous perception is based on the supposition that there are differences in the time taken to process different attributes. What I mean by processing time here is the time taken for activity at a given node (station) of the visual pathways to achieve a conscious correlate. The precise time taken, from onset of the stimulus to the moment of awareness of that stimulus and its characteristics, has not been measured. Perceptual asynchrony rather refers to the relative times taken to become aware of different visual attributes [10].

If the asynchronies are the result of differences in processing times (as defined above), then one should be able to manipulate the degree of asynchrony by varying the parameters of the attributes that are to be paired. Arnold & Clifford [18] were the first to provide direct evidence for this, by showing that the degree of motion–colour asynchrony can be reduced or increased by changing the angular difference in the direction of motion that is to be paired with colour. More recently, this has been shown to be true even within a single attribute, that of visual motion [19]. No asynchrony is detected in pairing experiments in which subjects are asked to pair up–down motion presented in one half of their field of view with left–right motion presented in the other half [9]. However, when asked to pair up–down motion with up–right motion, a temporal advantage in favour of up–right motion emerges. This can be accounted for by the fact that there is much less cortical inhibition for up versus up–right motion than for up–down versus left–right motion [19]. Motion in one direction suppresses or delays subsequent responses of cells in V5 to motion in the same or opposite direction [20,21]. Hence, the suppressive effects of up–down motion would be similar to those of left–right motion, and the pairing should result in no asynchrony. By contrast, there is much less suppression or delay between non-opponent motion directions (up and up–right); hence, the suppressive effects are relatively greater for up–down motion than for up–right motion, with the consequence that up–right motion is perceived first. Equally, it is well known that, although V5 responds to isoluminant stimuli that are in motion, the response is much attenuated [22,23]. Correspondingly, when the up–down motion of isoluminant dots is paired with the left–right motion of luminance dots (or vice versa), the advantage lies with the luminance dots [19], whereas no asynchrony is found when the same pairings are between luminous or between isoluminant stimuli. Hence, one can manipulate the asynchrony in a number of ways, which implies that it may indeed be the result of differences in processing times.

**Figure 3.**
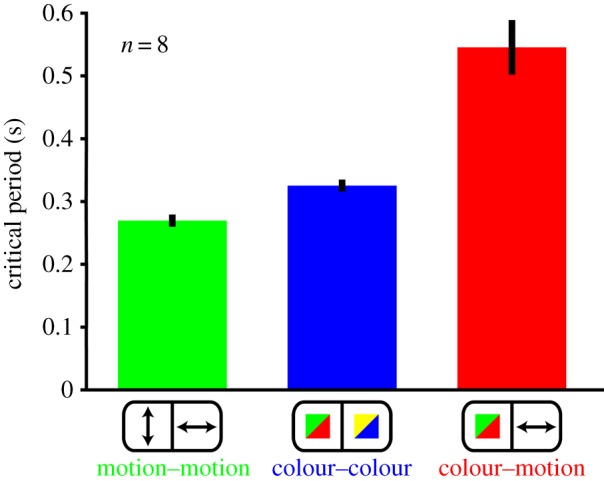
The results of psychophysical experiments to show the temporal difference in binding the same attribute (motion–motion, colour–colour) and different attributes (colour–motion) across the two hemi-fields. (Adapted from Bartels & Zeki [24].)

Perceptual asynchrony of a different temporal order can also be demonstrated when subjects are asked to pair similar (colour–colour; motion–motion) or different (colour–motion) attributes distributed across space (that is across the two hemi-fields, when the images would be projected to the two hemispheres separately). Now, motion–motion pairings take temporal precedence over colour–colour pairings which, in turn, take precedence over colour–motion pairings [24]. This may seem surprising, given the precedence in perceiving colour over motion, but is explicable by a difference in the conduction velocities of the fibres that unite areas V4 and V5, specialized for colour and motion respectively, in one hemisphere with their counterparts in the other; the interhemispheric connections of V5 are mediated by larger myelinated fibres than those mediating the interhemispheric connections of V4. This therefore also suggests that asynchronies can be traced to differences in processing times (see below and figure 3).

## 4. Temporal hierarchies in visual processing

Perceptual asynchrony introduces a new and unexpected element into visual processing, namely that of temporal hierarchies, which is distinct from the hierarchies in serial processing systems and not to be confused with them [10]. Perceptual temporal hierarchies may even be in reverse order from the one expected from serial hierarchical processing chains (see below). Perceptual asynchrony thus raises the question of how attributes that take different times to process and to perceive over brief time windows are bound together over longer periods to give us our apparently integrated perception of the visual world, where all the different attributes are seen in precise temporal and spatial registration (see [25,26] for excellent general reviews).

Functional specialization in the visual brain was demonstrated over 40 years ago [2,27–29] and immediately raised the question of how different attributes of the visual scene, processed in separate pathways, are bound together. Perceptual asynchrony was demonstrated 18 years ago [9]; it showed that we do not perceive all the attributes of the visual world at the same precise moment. This, in turn, immediately suggested that binding might be a more complex temporal issue than previously supposed and even raised questions about the nature of visual consciousness [30]. Yet, little attention has been given to perceptual asynchrony, which results from functional specialization, and its consequences. This is surprising. The binding problem was a central issue even before the demonstration of perceptual asynchrony and continues to be so; it even assumed a role in discussions of how conscious experiences are generated [31]. It is also surprising, because the problems raised, it seems to me, are huge and of importance in understanding how the brain functions. And the problem is one that is shared by those who believe in an extreme hierarchy or no hierarchies as much as by those who believe in parallel, modular, processing systems.

In this review, I do not discuss synchronization and binding between the activity of cells in a given area, registering the same attribute (e.g. orientation of lines). This *intra-attribute* binding may serve as a ‘glue’ that enables the brain to ‘bind’ the responses of cells located in geographically separate positions within the same visual area but which otherwise respond to the same attribute, for example the same orientation of lines. This is a topic that has been extensively addressed (see [32,33] for reviews), but is distinct from the one that I am addressing here, the question of *inter-attribute* binding. The temporal requirements for the two kinds of binding are probably significantly different. At any rate, the two types of binding should be distinguished, perhaps by referring specifically to *inter-* and *intra-attribute* binding.

## 5. Problems created by perceptual asynchrony

Perceptual asynchrony creates a problem for understanding how the visual brain operates, no matter what strategy one believes in.

For those who believe in ‘multiplex’ cells coding for colour, form and direction of motion [6] or for just colour and form [7,8], the problem is to account for some waiting mechanism in multiplex cells that regulates their responses to two or more attributes in time, to ensure that the final signal from the cell is that of an integrated output which signals colour, form and motion simultaneously; alternatively, they have to account for why, given perceptual asynchrony, the responses of such cells are perceptually ineffectual. Just how perceptually ineffective such cells are, assuming them to have the multiplex role imputed to them, is suggested by experiments on inter-attribute binding [34–36] which show that the accuracy of reporting two simultaneously presented attributes—colour and form—are independent, with accuracy for reporting the correct colour being higher than the one for reporting the correct form, presumably because colour is processed faster. This is so even when both attributes are focally attended to [36], a result that would not be expected if individual cells, wherever they may be located within the visual brain, code for both attributes.

Those subscribing to a hierarchical strategy within each of the parallel chains, with activity in a chain acquiring a conscious correlate only at the terminal node of the chain, assuming such a terminal node exists (see below), also face much the same problem. They have to account for some mechanism that waits until the final nodes in each of the parallel hierarchical chains terminate their processing and acquire a conscious correlate. That, over brief time windows, we misbind attributes that, veridically, occur at the same time [9] implies that there is no waiting mechanism in the brain, no universal visual clock which sets the time for all the processings in the terminal nodes of parallel hierarchical chains to terminate and acquire a conscious correlate.

Those who believe in parallel strategies, with activity at each node of a parallel chain capable of acquiring a conscious correlate [5], face the identical problem, but now magnified several times, because any hypothetical neural clock would have to wait for all the nodes within the parallel chains, not just the end nodes, to terminate their processings. In such a system, some kind of mechanism must be capable of binding activity at each node of each of the parallel systems to each node of any of the other parallel systems, a task of some magnitude.

### (a) Hierarchical processing systems

**Figure 4.**
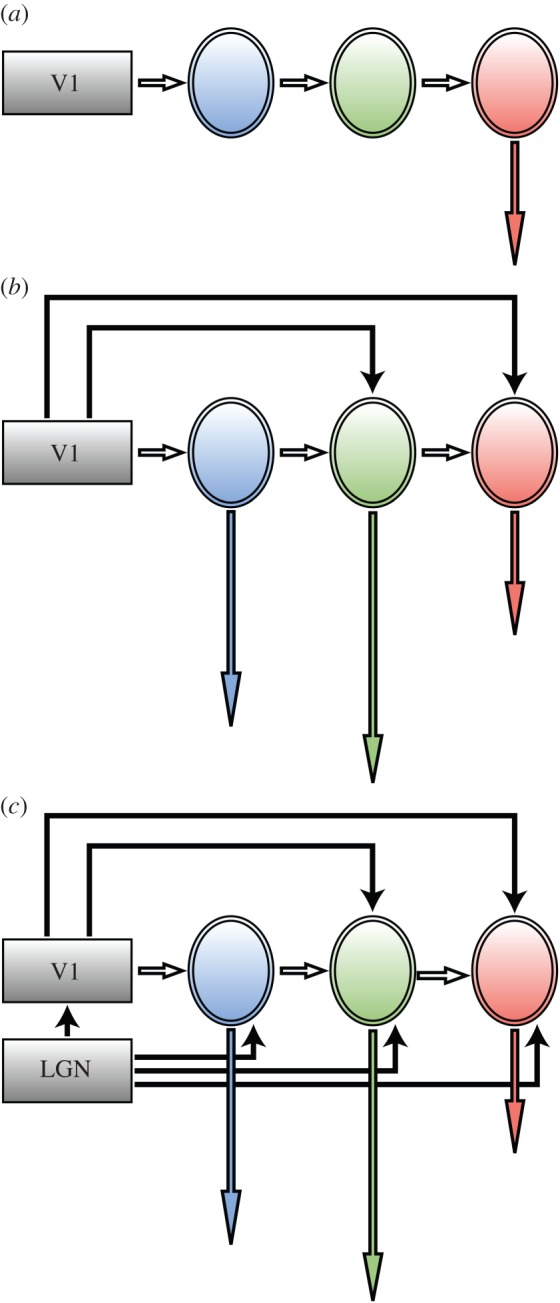
(*a*) The classical picture of a single hierarchical chain with two (or more) nodes and a terminal node (in pink) at which activity becomes perceptually explicit (needs no further processing). (*b*) The parallel outputs from V1 to two (or more) areas of the prestriate visual cortex and the serial, hierarchical, connections between them. Unlike in (*a*), activity becomes perceptually explicit at each node of the chain, the length of the downward arrows representing the asynchronous outputs from the nodes. (*c*) The more elaborate picture of parallel outputs from V1 and directly from the LGN–pulvinar to three or more areas of the prestriate cortex, as well as the serial connections between the visual areas. As in (*b*), the length of the downward arrows signifies that the outputs from these nodes are asynchronous with respect to each other.

There is little doubt of a hierarchical chain in anatomical connections, extending from the lateral geniculate nucleus (LGN) to the primary visual cortex (area V1) and from there to areas such as V2, V3, V4 and V5 of prestriate visual cortex (figure 4*b*). In addition, prestriate visual areas are connected to each other hierarchically, as in the examples of the connections from V1 to V2 to V4 in the colour system [37] and the connections from V1 to V2 to V5 in the motion system [38,39], thus introducing a hierarchical element into each of the parallel processing systems ([3,4] for reviews).

There is also impressive evidence in favour of a physiological hierarchy. The earliest came from the studies of Hubel and Wiesel, who showed a gradation in complexity of response between orientation-selective cells [40] within V1 itself [41,42], and between orientation-selective cells in V1 and those in areas of the prestriate cortex [43]. There is, as well, evidence of a functional hierarchy within the colour system, extending from V1 to V4 [44–47], with the responses of cells in V4 approximating more closely to constant colours and to specific hues than those in V1 and V2. Among the most impressive evidence in favour of a functional hierarchy is the one provided by the motion system [48,49], which shows that the cells of V1 projecting to V5 respond to the direction of motion of the components of a visual stimulus, whereas those in V5 commonly respond to its true, global direction of motion. This no doubt reflects the fact that the connections from V1 to V5 are highly convergent [50], although this convergent input, which operates to enlarge receptive fields, is rather subtle, because critical computational elements that distinguish between local and global motion are undertaken within subregions of the large receptive fields [51].

### (b) Problems raised by the hierarchical system

Hierarchical systems consisting of several stations, or nodes, within a chain raise operational and perceptual problems, although there may be other ones besides. If the sole function of a node A within a hierarchical chain is to process signals for the next stage, B, within that same system, then what has been processed in A will be lost to perception, which would represent a considerable waste of neural resources and is improbable [5] (figure 4*a*). More likely, what is processed at each node must be available to conscious perceptual experience, without further processing in subsequent nodes within the hierarchical chain (this is not to say that a node does not undertake other functions which may be important to the next node in the chain, for example that of error detection). In other words, activity at each node must, potentially, have a conscious correlate. But, if that is so, then the outputs from the nodes of a hierarchical system must themselves be parallel, because the output from node A could be independent of that from node B and vice versa (figure 4*b*). Parallel outputs imply a certain degree of autonomy of each node within a hierarchical chain, making each not totally dependent on either the previous node in the chain or the next one for activity in it to acquire a conscious correlate. This would constitute, therefore, one solution to the perceptual problem posed by an exclusively hierarchical system. There is indeed evidence to suggest that a parallel output system is used by the brain (see below). But, such a solution creates problems for the interbinding of visual attributes processed in separate areas of the visual brain, which I return to below.

One can give examples from all three visual systems discussed here, whether one is a proponent of the hierarchical or the parallel doctrine. In the form system, one can be aware of a complex form as well as of its constituent parts, including single oriented lines; if oriented lines are the preserve of early visual areas and complex forms of later areas, it follows that activity at each must become perceptually explicit. Although the colour system of the brain operates to confer a constant colour on objects and surfaces, in spite of wide-ranging fluctuations in the wavelength–energy composition of the light reflected from them [52], an individual can nevertheless be aware of sudden changes in wavelength composition (illumination) of the light [53]. It is likely that the former is signalled through the activity of cells in V4 [45,47,54,55], and the latter through the activity of cells in V1 and V2 [56,57], as well as some in V4—all three areas constituting hierarchical nodes within the brain's colour system. For the motion system, patients blinded by lesions in V1 but with an intact V5 can become aware of fast visual motion, whereas a patient rendered akinetopsic (motion-blind) [58] by a lesion in V5 but with an intact V1 can become aware of slow but not fast motion [59,60], again suggesting that activity at each of these two nodes can acquire a perceptual correlate, though a much impoverished one.

## 6. End nodes in the brain?

Based on anatomical evidence, I have argued in the past [61] that there is no end station in the cerebral cortex. This argument derives from the fact that all areas of the cerebral cortex have anatomical inputs and outputs, and receive and send signals. Consequently, there is no terminal station in the cerebral cortex. While this remains true in terms of anatomy, it requires reformulating in perceptual terms. If activity at each node of a chain can become perceptually explicit without the need for further processing, then it is reasonable to argue that, perceptually, there are end nodes in the brain, activity at which can be perceptually explicit without further processing. But, because, these nodes are recipient of feedback connections, which may modulate their activity, it follows that even such a modicum of perceptual independence does not constitute an endpoint. The critical issue here, which I return to below, is that these feedback inputs must themselves be acting asynchronously, thus highlighting further the asynchronous operations of the brain.

## 7. Parallel processing systems

**Figure 5.**
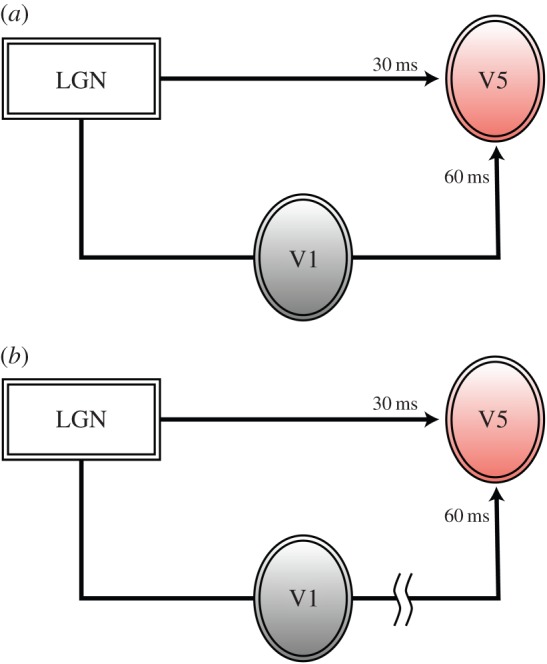
A simplified diagrammatic representation to show the asynchrony in the arrival of signals from two different sources, using V5 of prestriate cortex as an example. (*a*) The direct input from the LGN–pulvinar delivers signals to V5 at much earlier latencies (*ca* 30 ms), whereas the classical pathway through V1 delivers signals later (*ca* 60 ms). There is, as well, a difference in the motion signals delivered by these two pathways; the former delivers predominantly signals from fast-moving stimuli, whereas the latter delivers signals predominantly from slow-moving ones [68,69]. (*b*) When V5 is disconnected from V1 following a lesion in the latter, signals from the LGN–pulvinar can still reach V5, and the activity in the latter can become perceptually explicit, as in the Riddoch syndrome. All other details of the cortical connections of V5, including the return connections from V5 to V1, are omitted from this simplified diagram. (Online version in colour.)

The evidence for parallel processing, in both anatomical and physiological terms, is impressive. It is evident in the parallel outputs from each cortical visual area, including area V1, to other visual areas (figure 4*b*). But parallel processing is almost certainly a more extensively used strategy than previously supposed. It is now becoming apparent that a relative independence for the nodes constituting a chain within a parallel visual system is conferred on them not only by parallel cortical inputs originating in V1 and other visual areas, but also by parallel inputs from subcortical visual stations (figure 4*c*). While the major emphasis in research on the visual brain has revolved around the retina–LGN–V1 system, it has also been long known that both the LGN and the pulvinar project directly to visual areas of the prestriate cortex [62–66]. These projections have been relatively neglected in the past and their significance has only recently become evident, especially with reference to area V5 [67] (figure 5). This relative neglect may be due partly to the greater prominence of the LGN–V1 pathway, partly to the historical progression of research, during which activity of cells in V1 was charted before that in prestriate visual areas, and partly because of the latency of responses to flash visual stimulation in V1 and in prestriate cortex. This showed that, broadly, there is a temporal hierarchy in responses, consistent with a hierarchical view, with responses in V1 having shorter latencies and therefore preceding responses in prestriate cortex. Probably, all three reasons played a role. But, a reading of the evidence shows that there is significant overlap in response latencies obtained from V1 and visual areas of the prestriate cortex [70–72]. More recently, it has been found that, when stimuli tailored to the physiology of the visual areas, instead of flash stimuli, are used, the temporal order may not be in accord with the hierarchical order reflected in the connections from the LGN to V1 and from there to the specialized visual areas. With fast-moving visual stimuli, the latency of responses in V5 precedes those in V1 [68,69]. Thus, the input that reaches V5 from the subcortex in parallel with the input that reaches it from V1 also constitutes one of the best examples of parallelism in the visual brain. But the two parallel inputs do not deliver their signals synchronously; hence, V5 also provides one of the clearest examples to date of asynchronous parallel operations. There is evidence that this ‘V1-bypassing’ input can sustain a certain level of visual activity that has, as a correlate, a conscious visual experience. Patients blinded by lesions in V1 can have a rudimentary and much impoverished, though nevertheless conscious, experience of the direction of fast-moving visual stimuli without being aware of the other characteristics of the moving stimulus. [73]. Such patients can apparently also be aware of coloured stimuli if they are large [74].

Parallelism involving parallel inputs from V1 and direct V1-bypassing inputs to prestriate visual cortex is not unique to V5. In the form system, stimuli constituted from oriented lines (lines, angles, rhombuses) appear to activate both V1 and areas V2 and V3 of prestriate cortex within the same time frame (25–40 ms) [75,76]. More complex stimuli constituted from lines (faces and houses) also activate V1 and areas of the prestriate cortex critical for their perception within the same time window of 25–40 ms [77]. This stands in opposition to the common assumption that, in form perception, V1 is activated first and before areas outside it are activated. It follows that parallel processing is a very significant part of the strategy used by the visual brain to process visual signals, far more so than previously supposed (figure 4). It helps to confer a certain autonomy on the nodes within a hierarchical chain, because it implies that their responses are not entirely dependent on input from antecedent cortical nodes.

## 8. Relative autonomy of nodes

The above suggests a relative autonomy for nodes within a processing chain, worth discussing briefly for two reasons. The first is that it is unlikely that a node within a chain that is completely disconnected from other cortical areas, in terms of connections with them, and thus solely dependent on subcortical visual inputs, will be able to process signals to completion. But which inputs and outputs are mandatory is not known. There is evidence that some inputs to, and outputs from, a node may not be necessary for that node to process signals in a way that makes the endpoint of what they have processed accessible to conscious experience. Equally, there is evidence to suggest that a node such as V5, isolated from V1 by a lesion in it, may be active when visually stimulated without the subject being able to perceive or discriminate, consciously or unconsciously, the visual stimulus [78]. But, perhaps the clearest evidence for a relative autonomy of a node that is part of a hierarchical chain comes from studies of V5, which show that an input to it that bypasses V1 can, at least in some patients, confer a modest ability to experience, consciously, visual motion. As mentioned, the motion system extending from V1 to V5, both directly and through area V2 [38,39], is one of the best examples of a hierarchical chain, both anatomically and physiologically. It is natural to suppose, therefore, that activity in V5 is entirely dependent on the healthy functioning of area V1. But this turns out not to be so. Physiological activity in V5, though impoverished, can apparently be sustained by the pathways that reach V5 without passing through V1 [79,80], just as cells in V2 and V3 are reactive to appropriate visual stimulation in the absence of an input from V1, although with significantly reduced strength [81]. The V5 activity mediated by this direct subcortical input can evidently acquire a conscious correlate. This is reflected in the Riddoch syndrome, when patients blinded by lesions in V1 are able to perceive, consciously, fast but not slow motion [73,82,83]. (For the nature of this consciousness, see reviews [84–87].)

Critically, this autonomy is not only conferred on V5 by direct inputs to it from subcortical stations, but it is also independent of the feedback from V5 to V1 [83], hence calling into question the notion that, to acquire conscious status, signals in a specialized visual area must mandatorily be referred back to V1 [88,89]. All this is not to imply that the perception of visual motion of those deprived of a V1 is not very significantly impoverished; it of course is. But it shows that input from V1 and feedback to it are not essential for activity in V5 to lead to a crude but conscious experience of fast visual motion.

Finally, it is important to emphasize here that not all activity in V5 of an intact brain reaches conscious awareness, because motion information in a peripheral location of the field of view, though invisible to humans, can modulate activity in V5 [90]. These results, together with results reporting the relationship between single cell activity in V5 and perceptual decisions, suggest that a direct relationship between activity of V5 cells and (conscious) perception and decisions relating to motion may be the privilege of subpopulations of cells in V5 [91]. This is consistent with the notion of the ‘quantized’ nature of visual awareness [92], which posits that visual awareness may arise from the action of a limited number of cells in the visual cortex or, indeed, within a single visual area or node.

## 9. Multiple asynchronous processings within a cortical area

A brief review of the operations undertaken by a single area, V5, suggests that visual areas may undertake several operations asynchronously. Signals from the parallel inputs to V5, from V1 and from the subcortex (LGN–pulvinar), reach it asynchronously. Signals from fast-moving stimuli (more than 22° s^−1^) reach V5 with latencies of about 30 ms, whereas those from slowly moving stimuli (less than 5° s^−1^) reach it (through V1) at latencies of about 60 ms [68]. This temporal privileging of the fast motion input into V5 no doubt reflects the fact that, although the pathways to the cortex from the M, P and K systems are mixed within it [93,94], the M system probably constitutes the dominant one that inputs into V5. The asynchronous input into V5 from these two parallel systems is complemented by what are probably also asynchronous inputs into it from antecedent cortical visual areas, including V1 and V2. Smith *et al.* [95] report that responses of cells that signal component motion in V5 start about 6 ms earlier than cells that respond to pattern motion and that it takes about 50–75 ms for pattern motion cells to build up their selective profile, suggesting a temporal hierarchy. Seventy-five ms is considerably longer than the shortest latency activation recorded in V5 with fast-moving stimuli [68,96]. In the absence of a ‘clock’ in V5 that determines that processing in it starts only when all the signals reach it, it becomes reasonable to suppose that V5 processes signals reaching it at different times asynchronously, and that the activity produced by the fast input to V5 becomes perceptually explicit (i.e. requires no further processing) without support from the input from V1. While in Riddoch syndrome patients, the V1-bypassing input to V5 can result in a crude but conscious perception of the direction of motion of fast-moving stimuli without input from V1, it is likely that, in the normal brain, this V1-bypassing input is integrated into the temporally hierarchical elaboration of pattern motion cells in V5, which suggests a further asynchronous operation within it.

The above-mentioned time-based activation studies imply, theoretically at least, that the activity of cells detecting fast motion and driven by the V1-bypassing input may become perceptually explicit before the activity of cells that signal pattern motion, which are driven by V1 inputs. This alone makes it possible, and even likely, that V5 processes separate motion-related signals asynchronously. It is also possible that other stimulus-related features, for example motion in depth [97–99], are also processed asynchronously with respect to motion in the fronto-parallel plane. This would make of V5 an area that processes several distinct, but motion-related, signals separately, in parallel and asynchronously. Indeed, V5 may have subcomponents that are specialized for specific motion features such as optic flow [100], or a clustering (even if a relatively weak one) according to speed of motion [101,102], implying that different groupings in V5 may process signals relatively independently from each other, making parallel and asynchronous processing a probability.

I have concentrated largely on V5, because it is one of the most extensively studied visual areas. However, it is unlikely that V5 is unique in this; more likely other nodes in the visual pathways also process, in parallel and asynchronously, a variety of signals related to their specialization, but used for different ends in relation to that specialization.

Even if signals reach different visual areas in parallel and synchronously, it does not follow that they process them synchronously. In the visual form systems, signals apparently reach V1 and the prestriate areas (V2 and V3, or the visual areas critical for the perception of faces and houses) within the same time frame, as is shown by the parallel inputs to V1 and prestriate areas when subjects view perceptually simple and complex form stimuli [75,77]. But, just like V5, prestriate areas such as the ones enumerated above also handle signals that reach them through V1, which may be asynchronous with respect to the signals reaching them directly from the LGN and pulvinar, pointing to possible asynchronous operations within them. The relationship of synchronous–asynchronous parallel inputs to synchronous–asynchronous operations within individual areas has not been tested and merits future study.

## 10. Parallel outputs from different nodes

**Figure 6.**
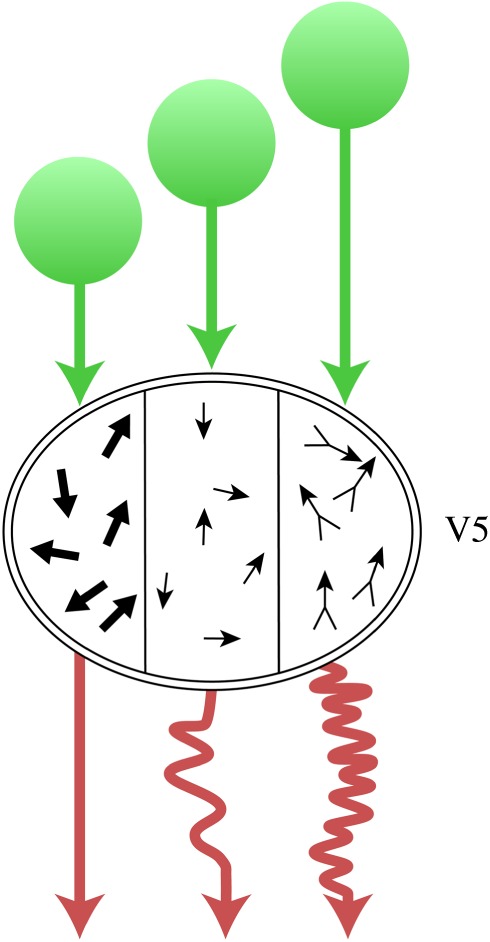
Diagram to illustrate the asynchronous operations within a single visual area (in this case V5), the asynchronous outputs resulting from the three operations (shown by differences in arrow lengths in red) and the asynchronous inputs from ‘higher’ areas to V5 (in green). The three compartments within V5 represent fast motion (left), slow motion (centre) and pattern motion (right). (Online version in colour.)

If an area undertakes several parallel operations asynchronously and in parallel (regardless of whether signals reach it synchronously or asynchronously), it is likely that the outputs from it will also be asynchronous (figure 6), unless one were to posit the existence of some clock within an area which dictates the timing of outputs from it. In the motion system, psychophysical experiments [19] have shown that different directions of motion are perceived asynchronously, suggesting that the activity of cells at single nodes, in this instance V5, may acquire a conscious correlate at a different time from that of other cells in the same node.

**Figure 7.**
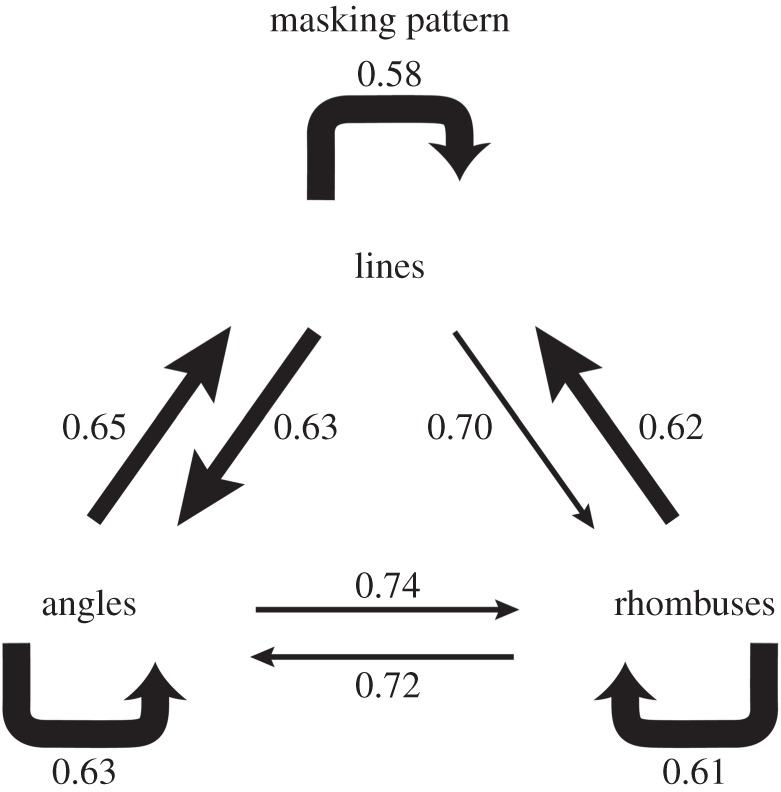
A summary diagram to illustrate the results of masking experiments in which subjects were presented with different target-mask pairs, using lines, angles and rhombuses. Heavy arrows show strong masking effects, whereas thin ones show weak effects. Lines are strongly masked by lines, angles by angles and rhombuses by rhombuses. In contrast, rhombuses are weakly masked by lines but lines are strongly masked by rhombuses. The numbers next to each line indicate the accuracy of target identification. (Adapted from Lo & Zeki [103].)

If two or more areas process their incoming signals asynchronously, it is possible and even likely that the outputs from them may also be asynchronous, hence compounding the number of asynchronous outputs. In the form system, parallel outputs are implied by the results of psychophysical masking experiments which show that rhombuses constituted from oriented lines, whereas easily masked by rhombuses, are relatively resistant to masking by oriented lines [103] (figure 7), quite contrary to what might be expected from the widely adhered view of a strictly hierarchical strategy in form processing whose source lies in the orientation-selective cells of V1. Such a hierarchical system would predict results in which lines would be more effective at masking rhombuses than rhombuses would be at masking lines. This is because the rhombuses in these experiments were constituted from the same oriented lines whose perception they masked. This suggests, again, that there are parallel subsystems even within what is traditionally considered to be a single hierarchical chain—the form system—with parallel output possibilities from them. Clinical evidence supports this, too, because agnosias for static forms need not be accompanied by agnosias for them when in motion [104], whereas agnosias for line drawings of objects need not be accompanied by agnosias for the objects themselves [105]. Together with the demonstration that one subdivision of the lateral occipital complex (LOC), heavily implicated in shape and object recognition [106], responds to shapes but not to orientation of lines, whereas another to orientation but not to shapes [107], these results argue against an interpretation of form construction solely in hierarchical terms, with the exclusive source of the hierarchy being the orientation-selective cells in V1. As well, parallel but asynchronous outputs from different nodes in the parallel visual processing systems are also strongly implied by the observation that the reaction times of human subjects to different visual attributes vary [12] and the observation that, even under conditions of focal attention, errors made in correctly identifying colour and form (orientation) are independent [36], presumably because colour is processed faster than orientation.

Such parallel outputs are also very much implied by significant differences in the activity time courses of different visual areas when subjects view action movies consisting of many different visual features, and hence activity in many specialized areas [108]. The activity time course between different visual areas decorrelates when viewing movies, compared with the resting state, the precise opposite of what one might have expected. This implies that the time courses of activity within different areas are not governed by a central clock but that activity at each is reset independently [109]—the precise opposite of what one might have expected.

## 11. Higher levels of perceptual asynchrony and parallel, asynchronous ‘top-down’ inputs to visual areas

More demanding visual tasks show that specializations for colour, form and motion are taken to further levels, because both the grouping of visual signals according to attribute and the formation of concepts according to them engage anatomically distinct parts of the parietal cortex [110–112]. Although the necessary experiments have yet to be done, it is intuitively reasonable to suppose that grouping according to colour will take temporal precedence over grouping according to motion, given that colour is perceived before motion. This raises the inter-attribute ‘binding’ problem at a yet higher level. Nor are parallelism and asynchrony to be thought of solely in terms of ‘elementary perception’ that is the result of feed-forward, ‘bottom-up’ processes. It may extend to ‘top-down’ processes, widely suspected to operate in the visual brain (figure 6). In one of the few papers to address the question of perceptual asynchrony in relation to brain *voxels* that code for both colour and motion, Seymour *et al.* [113] have used multivariate pattern analysis techniques in imaging experiments to show that there are conjunction units in the visual brain that code for both attributes. I have reservations about the interpretation they give to their results but these reservations are immaterial in the present context. What is interesting is their explanation for how such units, assuming them to represent actual cells, handle the asynchrony. They write of a feedback which would ‘gate’ their inputs, because ‘the timing of this gating might be important for adaptation of ‘double-duty’ units in V1 that are selective both for the color and the direction of motion of the stimulus' [113], an idea also present in Clifford's review [25]. Such a ‘feedback’ must, of course, take account of the asynchronous processing of colour and motion and be applied accordingly, thus highlighting once again the asynchronous nature of brain operations. As well, much discussed these days, is the error prediction system of the brain [114–116]. Such a system must be operating on perceptual systems that are themselves asynchronous in their operation. The ‘top-down’ system is often thought of as operating in the direction of ‘lower’ areas from ‘higher’ ones. But, there is also the possibility that it operates within individual nodes or stations [116].

If processing within one system, say the colour one, is faster than in another system, such as the motion system, it follows that the predictive coding operating on the two systems will be doing so asynchronously. Indeed, such iterative operations between ‘higher’ and ‘lower’ areas need not be restricted to the cortex. The LGN is known to project directly to both V4 [117] and V5 [118]. Given the asynchronous perception of colour and motion, all this implies that the results of action of different ‘top-down’ systems may not be applied simultaneously, because that would involve waiting for all the processing systems to complete their tasks. More likely, the massive error prediction system must itself act asynchronously.

It is interesting here to allude again to the temporal difference between inter- and intra-attribute binding. It would seem that the two do not occur synchronously, which suggests that asynchronous operations must be even more widespread.

## 12. A post-perceptual solution to inter-attribute binding?

Parallel and asynchronous outputs make the problem of interattribute binding even more emphatic. How to bind activity at so many different nodes, when processing speeds within and between them differ, as do the outputs from and return inputs to them? We had previously suggested that binding is a multi-stage activity, because it can involve any node within one processing system with any node in another [119]. But, perhaps it is simpler than that. One solution that has been recently proposed to this complex and apparently insurmountable problem is that inter-attribute binding is post-perceptual [36]; it does not occur by direct physiological interaction between cells in the specialized visual areas but post-perceptually, through fast-acting memory mechanisms, in the millisecond band, which are coincidence detectors. In such a system, the activity occurring at two nodes, no matter where they are located within the processing chains, would be bound if coincidence detectors detect these activities to have occurred within the same phase or cycle of some on-going activity. This may involve the hippocampus [120], in which temporal discontinuities may be bridged [121]. The merits of such a proposition have yet to be tested.

## 13. An overall strategy for visual processing

So how could we envision the brain solving a problem that is imposed by a natural world in which different visual attributes do not co-occur but covary instead, and one in which different attributes are processed at different speeds?

One solution is to use parallel processing. This became evident a long time ago, but the extent of its use by the brain is only now becoming apparent. Parallel connections in the brain have been much discussed and their computational significance assessed [122,123]. But, parallelism appears to be much more widespread than previously supposed. This is evident in the parallel inputs to a visual area not only from cortical, but also from subcortical sources. Moreover, parallel strategies appear to be used even within single processing systems such as the motion system, in terms of inputs to it and outputs from it and in terms of the parallel operations executed within it. The form system itself, almost universally considered to consist of a hierarchical chain commencing in V1, may consist of parallel subsystems, given that the orientation-selective cells of V1 may not be the sole source of inputs to the form system [75,76] just as, in the motion system, V1 cells responsive to motion are not the sole source of the input to V5.

Another strategy is to confer a certain degree of autonomy on each station or node of each parallel system, giving activity at each of its nodes the capacity to acquire a perceptual correlate without the necessity for further processing. But the imposition of such an autonomy, however limited, also raises the necessity of allowing for parallel outputs from the different nodes, with an output from one area not being necessarily totally dependent on either the previous area in the hierarchical chain or a subsequent one. Moreover, if the processing time (as defined above) at each node is not necessarily the same as the processing time at another or other nodes, then the outputs from different nodes must be asynchronous with respect to each other, as must the return inputs to them. It follows that the visual perceptual system is massively parallel, both spatially and temporally.

It also follows that even a relatively simple system restricted to the processing of form, colour and motion would require an extremely complex coordination in time.

## 14. A possible solution

The solution, I suggest, that evolution has adopted for the brain is to make of the visual brain a totally asynchronous organ, one in which the parallel systems operate with a fair degree of temporal autonomy and in which activity at different nodes of different parallel processing systems is not simultaneously reset to zero continuously by some master clock in the brain. If that is the solution, then it follows that binding of different visual attributes must itself be subject to a different rule than the one we have been entertaining so far [36]. One suggestion would be that discrete perceptual events occurring at different nodes are somehow related to another timing system, say, the theta rhythm in the hippocampus (6 Hz). Two or more events occurring within the hippocampal cycle might then be perceived as being bound. There is, indeed, some clinical evidence to suggest that this may be so [124,125]. Although there is no experimental evidence for this, it emphasizes the need to study neural events surrounding very short memories [31].

As an example, and entirely speculatively, let us suppose that visual area A has an ongoing alpha cycle and visual area B has another alpha cycle, not in phase with that of area A (whether separate visual areas have their own alpha cycles is not known). When the subject views a stimulus consisting of two attributes, say colour and motion, the desynchronization between the alpha activity in the two areas becomes maximal, since stimuli cause desynchronizations, the so-called evoked response desynchronization [126]. If alpha rhythms in area A and area B, which in this speculative argument are asynchronous with respect to each other, can nevertheless coincide in their cycle with the hippocampal theta rhythm or be related to it in some temporal way, signifying that the two events occurred concurrently, and are read as such by hippocampal ‘reader’ neurons [127], then the two separate events may be perceived as if bound. (Arnold [26] writes similarly of coincidence detectors.) In fact, it is known that there are unique firing patterns in the hippocampus that correlate with unique visual events (see [128] for a review). Hence, it is the relationship of two desynchronized rhythms to the rhythm in the hippocampus that becomes critical. It is evident that in such a system, no central clock is necessary. It is simply a question of one system ‘tagging’ onto another through a third. Whether this approaches what happens even remotely remains to be seen. This is not an entirely novel idea. Similar ideas have been proposed in the past [129], though not with reference to the visual brain or to binding different visual attributes. Nor is it necessarily the only solution. What seems to me to be necessary to emphasize in the future, far more than we have in the past, is the apparently ubiquitous asynchronous operations of the brain.

Computer technologists, who were relatively late in understanding the power of parallel systems [130] and who have never acknowledged the primacy of anatomists in uncovering the parallel operations of the visual brain [61], are now seeking to develop a new generation of computers that are faster and more efficient than the present generation of computers [1]. The defining feature of such computers is that they operate asynchronously.

The brain may, in fact, be much more like asynchronous computers than synchronous ones.

Or rather, asynchronous, parallel, computers may end up being much more like the brain than parallel, synchronous ones.
